# Demagnetization Fault Diagnosis of Permanent Magnet Synchronous Motors Based on Stator Current Signal Processing and Machine Learning Algorithms

**DOI:** 10.3390/s23041757

**Published:** 2023-02-04

**Authors:** Przemyslaw Pietrzak, Marcin Wolkiewicz

**Affiliations:** Department of Electrical Machines, Drives and Measurements, Wroclaw University of Science and Technology, Wybrzeze Wyspianskiego 27, 50-370 Wroclaw, Poland

**Keywords:** permanent magnet synchronous motor, fault diagnosis, condition monitoring, demagnetization, short-time Fourier transform, artificial intelligence, machine learning, neural networks

## Abstract

Reliable fault diagnosis and condition monitoring are essential for permanent magnet synchronous motor (PMSM) drive systems with high-reliability requirements. PMSMs can be subject to various types of damage during operation. Magnetic damage is a unique fault of PMSM and concerns the permanent magnet (PM) of the rotor. PM damage may be mechanical in nature or be related to the phenomenon of demagnetization. This article presents a machine learning (ML) based demagnetization fault diagnosis method for PMSM drives. The time-frequency domain analysis based on short-time Fourier transform (STFT) is applied in the process of PM fault feature extraction from the stator phase current signal. Moreover, two ML-based models are verified and compared in the process of the automatic fault detection of demagnetization fault. These models are k-nearest neighbors (KNN) and multiLayer perceptron (MLP). The influence of the input vector elements, key parameters and structures of the models used on their effectiveness is extensively analyzed. The results of the experimental verification confirm the very high effectiveness of the proposed method.

## 1. Introduction

Permanent magnet synchronous motors (PMSMs) have attracted much attention in the last twenty years in a wide range of applications such as sustainable energy wind power generation, robotics, electric vehicles, aerospace, industrial drives and many other fields. This is due to their high efficiency, high power density, excellent dynamic performance and simple, integrated design [1,2]. PMSMs also guarantee quiet operation, a high power factor and a long lifetime. This results in their widespread use in household appliances, HVAC and other commercial applications [3].

PMSM is a kind of motor in which permanent magnets (PMs) are installed on the rotor to provide excitation. Thanks to such a rotor design, there are no excitation losses caused by the flowing current in the rotor cage, compared, for example, to induction motors [4]. Despite many advantages, PMSMs, such as other electric motors, are exposed to various types of damages. These damages can be divided into electrical, mechanical and magnetic faults [5]. Electrical faults are mainly stator winding failures, in particular short-circuits, which are caused by insulation damage [6]. Among the mechanical damage, eccentricity and bearing damage are the most common [7].

Magnetic damage is a damage specific to PMSMs. It is related to the PMs of the rotor. Damage to PMs can be divided into mechanical damage of the PM (cracks, chipping) and those associated with the phenomenon of demagnetization [1,8]. There are many possible causes of demagnetization, such as excessive operating temperature, PM aging, severe flux weakening, and inter-turn short circuits (ITSCs) in stator winding [9]. In the case of the ITSC, a current with a very high amplitude flows in the shorted part of the winding, which causes local temperature increases. This is associated with the risk of exceeding the Curie temperature, and thus damage to the PM [10].

Additional exposures are also connected with the normal operation of the drive system. During the normal operation of the PMSM drive, the magnetic field generated in the stator winding opposes the magnetic field coming from the PM. This continuously repeated process, over time, especially in the case of overcurrent, gradually contributes to the demagnetization of the PMs [11]. Demagnetization not only disturbs the air-gap flux density symmetry, stator phase currents, voltages and the generated electromagnetic torque, but also increases the acoustic noises, vibrations and copper losses. This also results in a lower overall efficiency of the drive system. Moreover, the motor draws a higher current to keep the reference speed and load compared to a machine with undamaged magnets, which further raises the temperature of the stator winding.

In modern drive systems, the high reliability and stability of the system operation play a key role. Effective fault detection ensures safe operation, speed up of the maintenance process and decreases unexpected downtime and additional costs. Considering the growing popularity of PMSMs, predictive maintenance, fault diagnosis and condition monitoring of these machines have also become very important [12]. In the case of PMSM drives, the diagnosis of PM faults is especially important, taking into account the negative effects associated with demagnetization.

Over the years, several methods have been developed for the diagnosis of the PMSM demagnetization [13]. Most of them are based on the processed diagnostic signals such as axial flux, back electromotive force (EMF), vibration and stator phase current. Signal processing methods allow the extraction of the characteristic fault features. These methods can be divided into frequency and time-frequency domain analysis. Among the methods that carry out the analysis in frequency domain, spectral analysis of the signal using the fast Fourier transform (FFT) is dominant. It consists in the analysis of increases in the amplitudes of frequency components characteristic of PM damage [14,15]. The zero sequence voltage components (ZSVCs) FFT analysis was proposed in [16] for the early detection of the PMSM rotor demagnetization. It was shown that local demagnetization reduces the amplitude of the ZSVC and this may enable fault identification. However, it is limited by the need to provide access to the neutral point of the stator windings. The other diagnostic signals combined with FFT are axial flux [17], back EMF [18] and vibration [19]. However, the FFT-based demagnetization fault diagnosis methods have also some limitations. The main limitation is the fact that after the processing of the time-domain signal using the FFT analysis, information about the time of occurrence of a given frequency component is lost. In addition, a long measurement of time is needed to achieve sufficient frequency resolution and symptoms extraction effectiveness. These limitations are devoid of methods that realize time-frequency analysis [20].

The result of signal processing realized by time-frequency domain methods contains information about the time of the frequency components occurrence. This is an important advantage, especially in the field of fault diagnosis, as it can allow one to determine the potential cause of the failure at a later stage. Time-frequency domain methods include, among others, the continuous wavelet transform [21], Hilbert–Huang transform [22], Wigner–Ville distribution [23] and short-time Fourier transform (STFT). The use of STFT analysis has not been studied in detail for use in fault diagnosis of PMSM demagnetization, especially in combination with methods that allow automation of the fault diagnosis process. Another, novel method used for the localization of the PM damage that is based on the analysis of the output signals of three toroidal yoke coils wound around the stator yoke is proposed in [24]. Vold–Kalman filtering order tracking is employed for the real-time rotor demagnetization fault features extraction from the torque ripples in [25].

All of the above-mentioned methods can be successfully applied for the symptoms extraction of the PMSM demagnetization fault. However, this is not sufficient for modern fault diagnosis systems. The idea of industry 4.0 also involves requirements for full automation of various processes, including predictive maintenance, condition monitoring and fault detection. Machine learning (ML) algorithms can be used to meet these requirements.

There have been several studies on fault detectors that are based on ML learning algorithms. In general, they can be divided into classical ML algorithms and artificial neural networks (ANNs), with both shallow and deep structures. In the diagnosis of PMSM demagnetization fault, the use of ANNs has been most often analyzed in the literature. The largest amount of research related to the development of PMSM fault detectors refer to the application of the NN with shallow structure-feedforward multiLayer perceptron (MLP). It is proposed for the PM demagnetization fault detection, among others, in [26]. In [27] the possibility of detecting this type of fault using a self-organizing Kohonen map trained with data obtained from the finite element method (FEM) based on the PMSM model is investigated. ITSC and demagnetization fault diagnosis strategy based on a self-attention-based severity estimation network is proposed in [28].

In recent years, increasing attention has been paid to the application of deep neural networks (DNNs) and, in particular, convolutional neural networks (CNNs) in fault diagnosis of electric motors. In [11], the PM damage fault diagnosis method based on the raw signal analysis combined with the CNN model is proposed. The demagnetization fault diagnosis method based on the analysis of a stator phase current combined with CNN is proposed in [29]. However, with signal preprocessing stage used in the fault diagnosis process, classic ML algorithms such as k-nearest neighbors (KNN) may be sufficient. Nevertheless, they have been extensively studied in the past for their applicability to induction motors and PMSM electrical faults diagnosis [30,31,32], rather than to detect PMSM demagnetization.

This paper proposes an application of STFT analysis of the stator phase current signal to extract PM damage symptoms in PMSM drives and compares two ML algorithms: KNN and MLP for the automatic detection of this type of damage. The main contributions of this research are as follows:

(1) Evaluation of the applicability of STFT analysis of the stator phase current signal to extract PM damage symptoms in PMSM drives, based on experimental tests performed under different operating conditions of the drive system.
(2) Determination of the fault features that are the most sensitive to PM damage, being at the same time the least dependent on motor operating conditions.
(3) Development of the hybrid diagnostic method combining STFT analysis and ML-based models: KNN and MLP for PM fault detection in PMSM drives.
(4) Detailed verification of the input vector elements, key parameters and structure of selected ML algorithms on the PM fault detectors effectiveness.
(5) Comparison of the effectiveness of KNN- and MLP-based PM fault detector models, and proving that the use of a simple KNN algorithm is sufficient to achieve very high detection effectiveness while maintaining a significantly shorter model response time compared to MLP.

The paper consists of six sections. Following this introduction, Section 2 describes the details of the physical modeling of the PMSM rotor PM damage and the impact of this failure on the stator phase current waveforms. Section 3 is devoted to the theoretical basis of the STFT analysis. Section 4 describes the experimental setup used in this research. The next section presents the STFT-based PM damage extraction part. The training process and experimental verification of the effectiveness of ML-based PM fault detectors are presented in Section 5. The paper ends with a conclusion section.

## 2. Impact of the PM Damage on the PMSM Drive Stator Phase Current Waveforms

The theoretical analysis, including circuit- and FEM-based mathematical models of the PMSM with demagnetization fault has been raised many times in the past in the literature [27,33,34,35]. This study focuses on the impact of PM damage on the stator phase current and on the extraction of symptoms of these faults from this diagnostic signal.

### 2.1. Physical Modeling of PMSM Rotor PM Damage

PMSM demagnetization faults can be physically modeled by removing parts of the magnets (mechanical damage) [36], the installation of weaker PMs in the rotor through manufacturers [37] or by heat treatment [26]. In the case of the heat treatment the PMs have to be removed to avoid the situation when a very high temperature would damage a greater number of PMs caused by thermal inertia. The PM demagnetization performed by heat treatment is also associated with difficulties in accurately determining the degree of PM damage [11]. In the first approach, mechanical damage to the PM is not subject to these limitations and has been realized, among others, in [11,27,38]. The PM damage of the PMSM rotor used in this study was also implemented according to this method.

**Figure 1:**
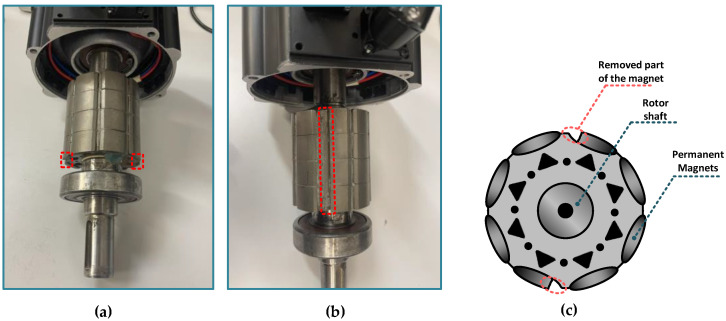
PMSM rotor with damaged PM: (**a**) construction of the tested PMSM rotor, (**b**) side view of the damaged rotor, (**c**) illustrative front of the rotor with implemented PM fault.

The construction of the rotor of the tested PMSM is shown in Figure 1a. The part of the magnets of one pole pair corresponding to ≈20% of a single PM area was removed along the rotor on opposite sides to implement partial demagnetization and to eliminate the unwanted effect of PMSM rotor unbalance, as indicated by red dashed boxes in Figure 1a. A side view of the rotor with a marked removed part of one pole is shown in Figure 1b. An illustration of the front of the rotor with implemented PM fault is presented in Figure 1c. Considering a motor with four pole pairs, it can be assumed that about 5% of the demagnetization is modeled for the tested PMSM.

### 2.2. Analysis of Stator Phase Current Waveforms and Its FFT Spectrum

**Figure 2:**
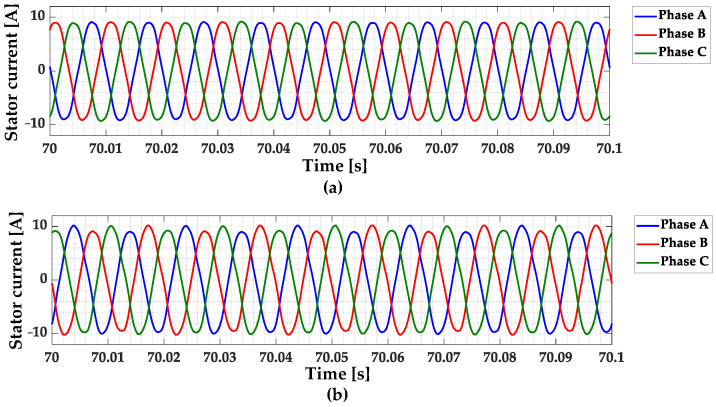
Stator phase current waveforms for (**a**) an undamaged rotor and (**b**) rotor with damaged PMs (partial demagnetization), experimental study (*T_L_* = *T_N_*, *f_s_* = *f_sN_* = 100 Hz).

**Figure 3:**
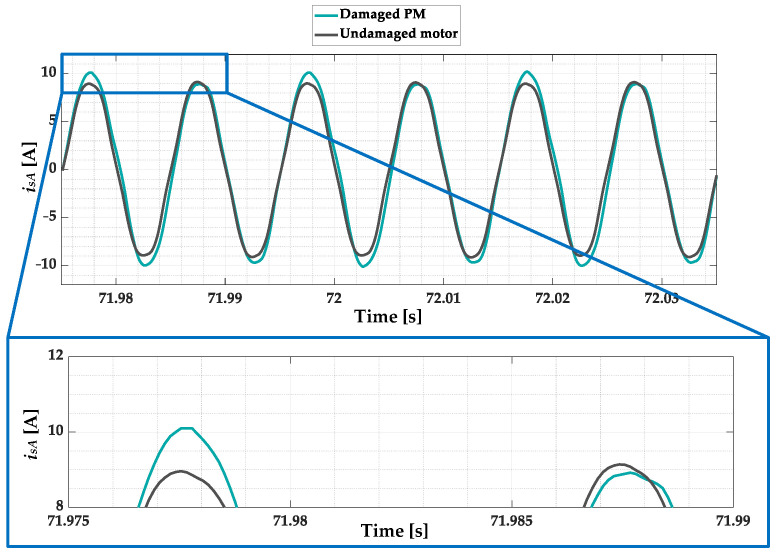
Comparison of the phase A stator current waveform for an undamaged rotor and rotor with damaged PMs (partial demagnetization), experimental study (*T_L_* = *T_N_*, *f_s_* = *f_sN_* = 100 Hz).

In the case of PMSMs, the source of the rotor magnetic field is the PM. Regardless of the type of damage to the magnets, the strength of the rotor (PM) flux decreases as a result of demagnetization. Since the electromagnetic torque of PMSMs is proportional to the cross-product of the current vector and PM flux linkage, the motor needs more (larger) current to maintain the same load torque level when demagnetization has occurred compared to an undamaged machine. In addition, the periodically repeated disturbances of the PM magnetic field introduced by the PM damage causes distortions in the induced back EMF. As a result of the appearance of these distortions, the shape and amplitude of the stator phase current waveforms are also affected. The stator phase current waveforms for an undamaged rotor and rotor with damaged PMs (≈5% of demagnetization) during motor operation at nominal speed and nominal load torque are shown in Figure 2. A direct comparison of a phase A stator current waveform for an undamaged rotor and rotor with a PM fault is presented in Figure 3. The analysis of the waveforms presented in these figures shows that the PM damage causes fluctuations in the amplitudes of the stator phase currents and a slight distortion of their sinusoidal waveforms. This is due to the additional harmonics that are included in these waveforms as a result of partial demagnetization.

To isolate the stator phase current waveforms changes caused by the PM damage, signal preprocessing methods are utilized. The classic approach to extracting PMSM demagnetization symptoms is based on the FFT analysis of the stator phase currents. The frequencies of the spectral components whose amplitudes increase as a result of PM damage are calculated as follows [11,39]:

(1) $$f_{PMDamage}=f_{s}\left(1\pm \frac{k}{p_{p}}\right)=f_{s}\pm kf_{r}$$

where: *f_s_*—fundamental frequency of the supply voltage, *f_r_*—rotational frequency, *p_p_*—number of pole pairs, *k*—consecutive positive integers (1, 2, 3…).

**Figure 4:**
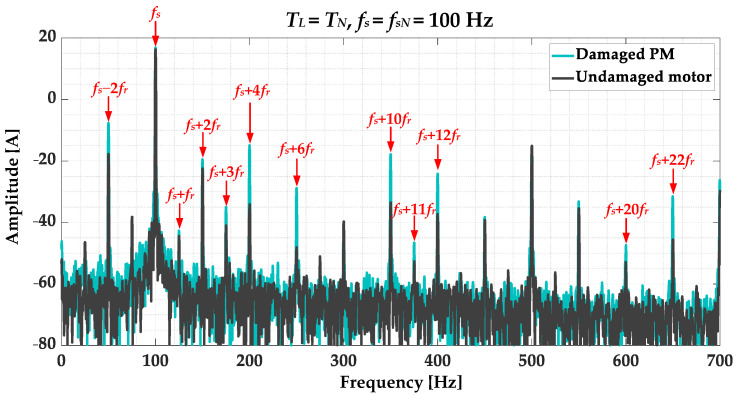
Comparison of the FFT spectra of the stator phase current in phase A for undamaged motor and rotor with damaged PM (partial demagnetization), experimental study (*T_L_* = *T_N_*, *f_s_* = *f_sN_* = 100 Hz).

The FFT spectra of the stator phase current in phase A for the undamaged PMSM and the rotor with the discussed damaged to the PM for nominal operating conditions are shown in Figure 4. Based on the comparison of these spectra it can be concluded that the amplitude increase in the frequencies described by Equation (1) is visible, especially for the *k* = {−2; 4; 6; 10; 12; 22}. For this reason, changes in the amplitudes of these harmonics will be especially tracked in the STFT analysis step described later in this paper.

## 3. Short-Time Fourier Transform Theoretical Basis

Information about the time of failure may be very important in the field of electric motor fault diagnosis. Based on this information, a potential cause of the failure can be found. While the result of FFT analysis does not contain information about the time of occurrence of a given frequency component, the result of STFT analysis retains this important information. STFT is also suitable for analyzing non-stationary signals [40].

The principle of STFT is based on dividing a signal in the time domain into windows of the same width, and then the frequency content of each of these windows is calculated using the FFT. The size of the window defines the time and frequency resolution of the STFT analysis. The shorter the window, the better the resolution in the time domain and the worse in the frequency domain [41].

STFT calculates the FFT of a function over a symmetric window function *w*(*t*), which is translated by time *t* and modulated at frequency *ω*, according to the following equation [42]:

(2) $$S(t,\omega )=\int_{-\infty }^{\infty }f(t)w(\tau -t)e^{-j\omega \tau }d\tau$$

The result of STFT analysis is a spectrogram of the signal. It is a three-dimensional plot of the energy of the frequency content of a signal as it changes over time. It is expressed as follows:

(3) $$\mathrm{spectrogram}(t,\omega )=|S(t,\omega ){|}^{2}$$

In real implementations, signals are sampled at a fixed sampling frequency (*f_p_*), Therefore, Equation (2) in the discrete domain can be expressed by the following equation:

(4) $$S_{D}[m,k]=\sum_{n=0}^{n=N-1}x[n]w[n-mH]e^{-j\frac{2\pi nk}{N}}$$

where: *N*—number of FFT points, *n*—time-domain input sample index, *x*[*n*]—input sample, *w*[*n*]—window function, *H*—window size (width), *k*—frequency index.

STFT analysis requires defining its key parameters at the algorithm design. These parameters include sampling frequency *f_p_*, number of input samples *N_t_*, window size *H* and type of window function *w*[*n*]. Their detailed description and analysis of the impact of selected parameters on STFT results can be found, among others, in [20,42].

## 4. Experimental Setup

**Figure 5:**
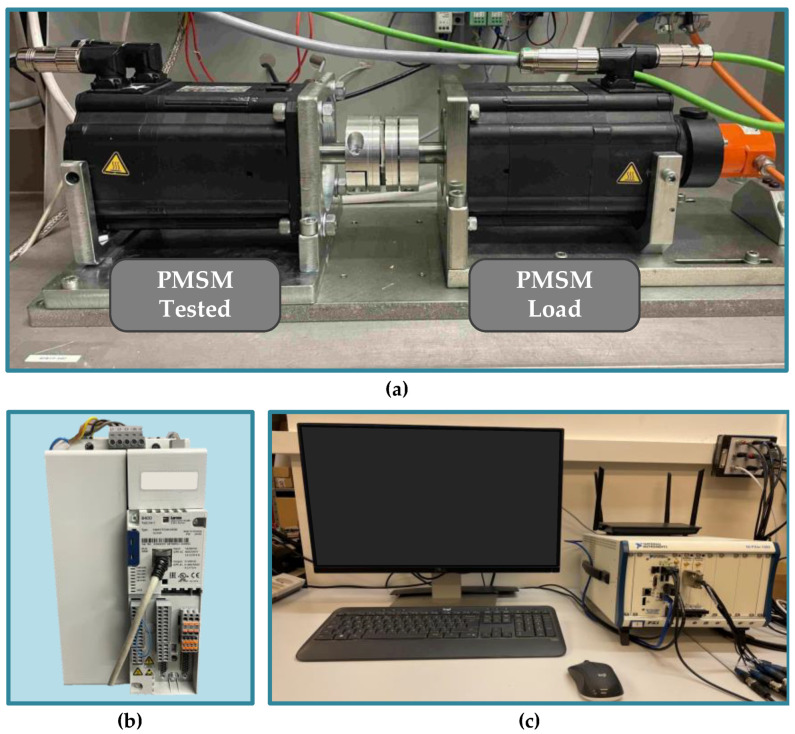
Real view of the experimental setup (**a**) motor test stand, (**b**) inverter Lenze 8400 Topline, (**c**) data acquisition system—industrial PC NI PXI 1082 (National Instruments, Austin, TX, USA) with NI DAQ card PXI-4492 (National Instruments, Austin, TX, USA).

The real view of the motor test stand is presented in Figure 5a. The main part is a 2.5 kW PMSM supplied from a Topline 8400 voltage source inverter (VSI) by Lenze (Lenze, Aerzen, Germany) (Figure 5b) and operating in the field-oriented control (FOC). This motor is tested with an undamaged rotor inside and also a rotor with a damaged PM. Details on the physical modeling of partial demagnetization are discussed in Section 2. The rated parameters of the tested motor are grouped in Table A1 in Appendix A. The load for the tested motor is provided by a second PMSM with a higher rated power of 4.7 kW.

**Figure 6:**
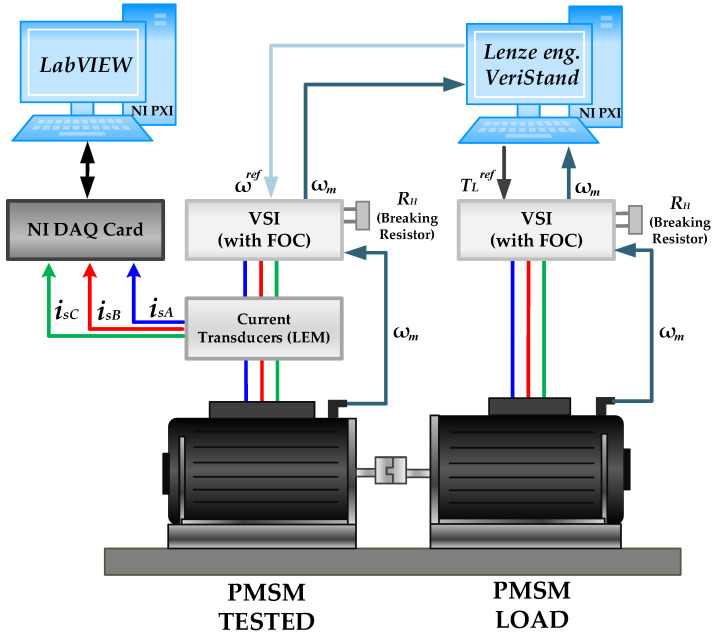
Block diagram of the experimental setup.

Analyzed diagnostic signals (stator phase currents) are measured with LEM LA 25-NP (LEM, Meyrin, Switzerland) multi-range current. These signals are then transferred to a data acquisition system. The data acquisition system consists of a national instruments (NI) DAQ NI PXI-4492 measurement card, with a 24-bit resolution A/D converter. It is housed inside an industrial PC (NI PXI 1082) (Figure 5c) with a NI PXI-e-8400 (National Instruments, Austin, TX, USA) quad-core embedded controller based on Intel Core i7-5700EQ processor and 4GB of RAM. The diagnostic application is developed using LabVIEW and MATLAB software. The Lenze engineer software is used to control the tested PMSM, while the VeriStand software is used to set the load torque of the loading motor. The block diagram of the experimental setup is presented in Figure 6. The tests are carried out for various values of the load torque in range *T_L_* = (0 ÷ 1)*T_N_*, with the step of 0.2*T_N_*, and various power supply frequencies.

## 5. STFT Based Extraction of the PMSM Rotor Permanent Magnet Damage Symptoms

In the scope of this research, extraction of the symptoms of damage of the PM magnet (partial demagnetization) is carried out by means of STFT analysis of the stator phase current signal. The amplitude level of selected harmonics can be useful information about the condition of the rotor. Therefore, the changes in the amplitudes of the frequencies characteristic of rotor PM damage will be analyzed in this section.

**Figure 7:**
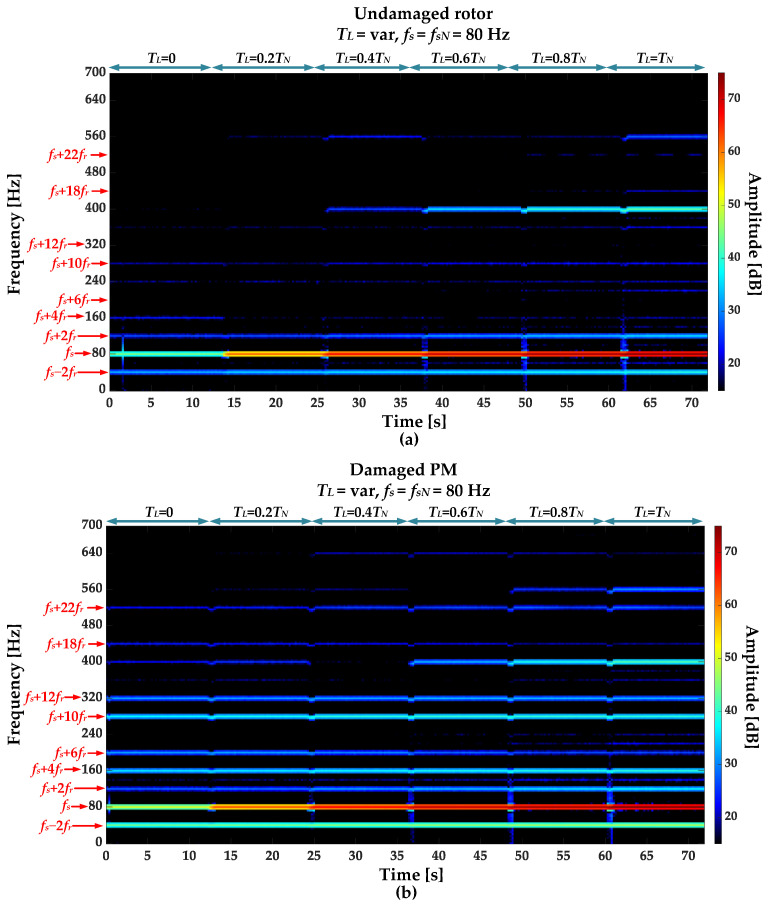
STFT spectrograms of the stator phase current component for (**a**) an undamaged rotor and (**b**) rotor with damaged PMs (*T_L_* = var, *f_s_* = 80 Hz).

**Figure 8:**
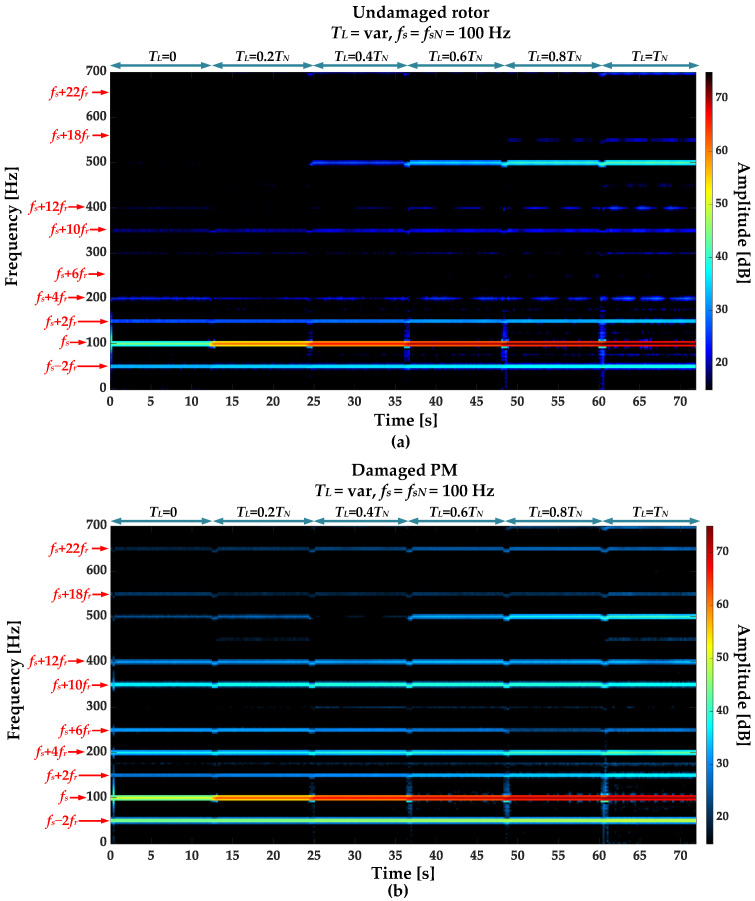
STFT spectrograms of the stator phase current component for (**a**) an undamaged rotor and (**b**) rotor with damaged PMs (*T_L_* = var, *f_s_* = *f_sN_* = 100 Hz).

STFT spectrograms of the stator phase current for an undamaged motor and a motor with a damaged PM of the rotor, operating with different set load torques, *f_s_* = 80 Hz and window width of 2048 samples, corresponding to a data collection time of 0.25 s, are shown in Figure 7a,b. STFT spectrograms for the nominal power supply frequency (*f_s_* = *f_s_* = 100 Hz) for the same rotor conditions are presented in Figure 8. For both cases, the spectrograms show an increase in the amplitude values of the frequency components described by Equation (1) for the *k* = {−2; 2; 4; 6; 10; 12; 18; 22}.

**Figure 9:**
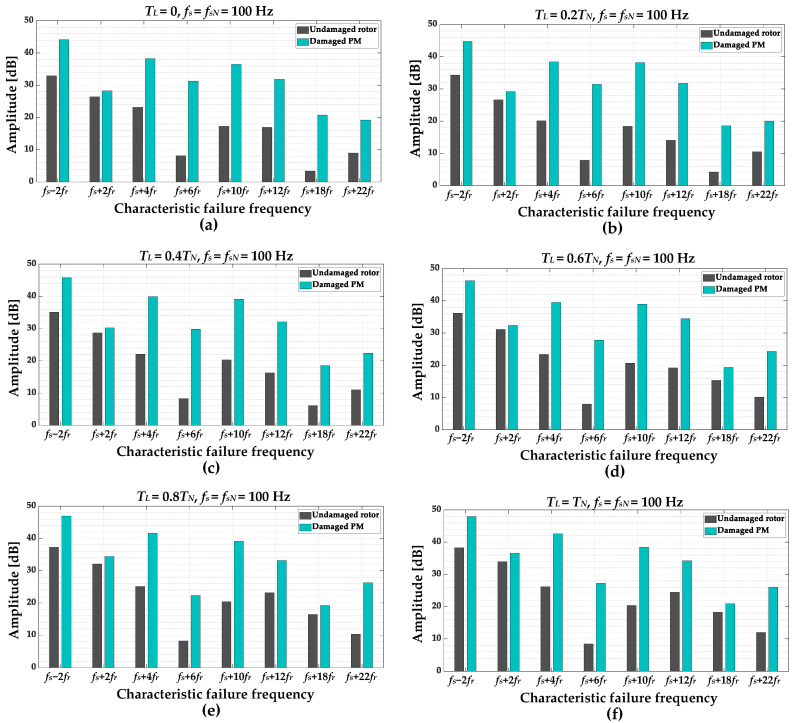
The impact of the damaged PM (partial demagnetization) of the PMSM rotor and *T_L_* on the amplitude levels of the frequency component in the stator phase current STFT spectrogram (**a**) *T_L_* = 0, (**b**) *T_L_* = 0.2*T_N_*, (**c**) *T_L_* = 0.4*T_N_*, (**d**) *T_L_* = 0.6*T_N_*, (**e**) *T_L_* = 0.8*T_N_*, (f) *T_L_* = *T_N_*.

**Figure 10:**
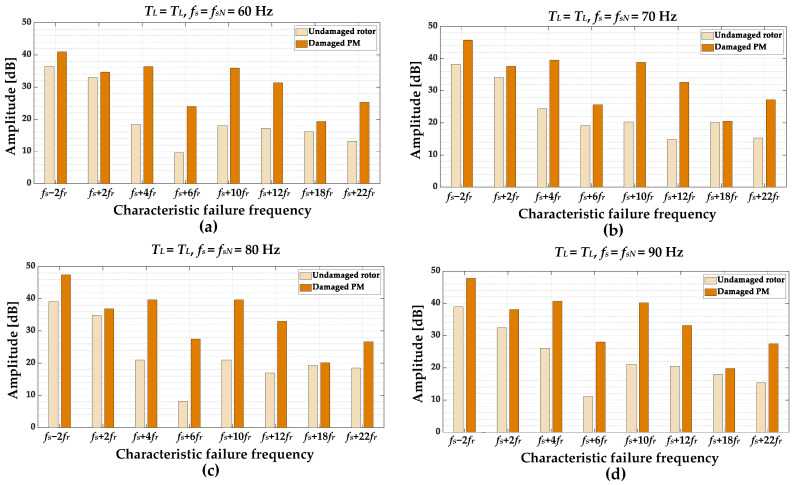
The impact of the damaged PM (partial demagnetization) of the PMSM rotor and *f_s_* on the amplitude levels of the frequency component in the stator phase current STFT spectrogram (**a**) *f_s_* = 60 Hz, (**b**) *f_s_* = 70 Hz, (**c**) *f_s_* = 80 Hz, (**d**) *f_s_* = 90 Hz.

In order to select the frequency components that are the most sensitive to rotor PM damage, the amplitude values of individual harmonics are compared for an undamaged motor and motor with a partially demagnetized rotor. The comparison is performed based on the experimental verification carried out in a wide range of motor operating conditions. The amplitudes comparison for different values of the load torque level *T_L_* in range *T_L_* = (0 ÷ 1)*T_N_*, with the step of 0.2 *T_N_*, and nominal power supply frequency *f_s_* = *f_sN_* = 100 Hz is shown in Figure 9. The influence of the *f_s_* value is illustrated in Figure 10. Based on the analysis of these results it can be inferred that the amplitude of each of the selected frequency components increases as a result of PM damage. For a more thorough analysis, in the next step, only the increases in individual frequency components as a result of partial demagnetization are analyzed.

In order to evaluate only the effect of PM magnet damage on the amplitude level of a given frequency component and to compare the increases between different harmonics, the amplitude increase caused by the PM damage in relation to the value for an undamaged rotor is analyzed:

(5) $$A_{DIFF}(f_{PMDamage})=A_{Damaged}(f_{PMDamage})-A_{Undamaged}(f_{PMDamage}),$$

where: *f_PMDamage_*—characteristic frequency component for PM damage calculated according to Equation (1) for different *k*, *A_Damaged_*—amplitude of the *f_PMDamage_* component for damaged rotor PM, *A_Undamaged_*—amplitude of the *f_PMDamage_* component for undamaged rotor.

The amplitude increases (differences between the amplitude value for damaged and undamaged rotor) of the selected frequency components characteristic of the PM fault together with the average difference *A_DIFFAvg_* and standard deviation *σ* for different load torques set and power supply frequencies are grouped in Table 1 and Table 2, respectively.

On the basis of the presented results, it can be concluded that the highest increases in the amplitudes caused by PMSM rotor PM damage are visible for the following frequency components in the stator phase current STFT spectrogram: *f_s_* + 4*f_r_*, *f_s_* + 6*f_r_*, *f_s_* + 10*f_r_* and *f_s_* + 12*f_r_*. The standard deviation of these increases for different motor operating conditions is also low, which allows us to conclude that they are not very sensitive to changes in motor operating conditions (*T_L_* and *f_s_*). Nevertheless, it would be difficult to manually define a single threshold value for the amplitude of selected characteristic frequency components, valid over a wide range of PMSM drive operating conditions, the exceeding of which would indicate PM damage. In addition, such approach does not allow generalization for different motor operating conditions. Therefore, in the next step, the analysis of the possibility of using these amplitudes as input vector elements of the ML-based PM fault detectors of PM to automate the fault detection process will be conducted.

## 6. Machine Learning Based Detectors of the PMSM Demagnetization Fault

Automating the condition monitoring and fault diagnosis process is critical for modern drive systems. This study proposes and compares two ML-based models for the rotor PM fault (partial demagnetization) detection of a PMSM drive. The analyzed models are a simple ML algorithm KNN and a shallow neural network MLP.

### 6.1. Theoretical Basics

#### 6.1.1. KNN

In the field of data classification, the KNN algorithm is considered one of the most fundamental ML algorithms [43,44]. The principle of the KNN algorithm is based on calculating the distance between a new data point and points that were involved in the training process (training data set). The new point is then assigned to the class to which the most points from its neighborhood belong. The number of neighboring points *K* (nearest neighbors) has to be determined at the model design stage [45,46].

The choice of *K* value has a major impact on the final accuracy of the classifier model [30,37]. Nevertheless, there is no specific definition of how to determine the *K* value. Therefore, it is necessary to check the results for different values to find the best one. This is often overlooked in many works that apply this model for fault diagnosis purposes.

At the stage of designing a classifier model based on the KNN algorithm, it is also necessary to choose a suitable function for calculating the distance between neighboring points. In the literature, various distance metrics are proposed [45]. Let *A* and *B* be a feature vectors: *A* = (*x*_1_, *x*_2_,…, *x_n_*) and *B* = (*y*_1_, *y*_2_,…, *y_n_*), where *n* is the feature space dimensionality. The most popular functions that have been used in the past to calculate the distance are the Euclidean, Minkowski, Mahalanobis and correlation functions. They are expressed by Equations (6)–(9), respectively. Among them, the most popular is the Minkowski distance metric [47]. The details of the KNN algorithm are discussed by the authors in [30,43,44,45,46,47].

(6) $$d_{Euclidean}(A,B)=\sqrt{\sum_{i=1}^{n}(x_{i}-y_{i}{)}^{2}}$$

(7) $$d_{Minkowski}(A,B)={\left(\sum_{i=1}^{n}|x_{i}-y_{i}{|}^{r}\right)}^{\frac{1}{r}}$$

(8) $$d_{Mahalanobis}(A,B)=\sqrt{{\left(\frac{x_{1}-y_{1}}{\sigma_{1}}\right)}^{2}+{\left(\frac{x_{2}-y_{2}}{\sigma_{2}}\right)}^{2}}$$

(9) $$d_{Correlation}(A,B)=\frac{\sum_{i=1}^{n}(x_{i}-\mu_{i})(y_{i}-\mu_{i})}{\sqrt{\sum_{i=1}^{n}(x_{i}-\mu_{i}{)}^{2}\sum_{i=1}^{n}(y_{i}-\mu_{i}{)}^{2}}}$$

#### 6.1.2. MLP

Among all types of neural network structures, one of the most widely used in the field of fault diagnosis is the MLP. MLP is characterized by a simple structure that includes an input layer, one or more hidden layers and an output layer. A characteristic feature of this type of neural network is that each neuron of a layer is connected to each neuron of the next layer. Compared to DNNs, MLPs are also characterized by simplicity of implementation.

At the stage of designing a data classifier, which is based on the MLP model, it is necessary to determine its structure (number of hidden layers, number of neurons in each layer) and type of activation function. The output of an exemplary MLP model with two hidden layers can be expressed by the following equation:

(10) $$y_{k}=f^{\left(2\right)}(\sum_{m=1}^{M}w_{km}^{\left(2\right)}f^{\left(1\right)}(\sum_{n=1}^{N}w_{mn}^{\left(1\right)}x_{n}+w_{m0}^{\left(1\right)})+w_{k0}^{\left(2\right)})$$

where: *x_n_*—*n*-th value of the input, *y_k_*—output value of the *k*—th neuron, *f*^(1)^, *f*^(2)^—activation function of the first and second layer, *w*-weight of the neuron in the selected layer.

The process of training the MLP involves modifying the weights to minimize the objective function [48]. The most commonly used algorithm in the MLP model training process is the Levenberg–Marquardt algorithm.

### 6.2. Development of the PMSM Demagnetization Fault Detectors

Based on the STFT analysis of the stator phase current, four elements are initially applied as the input vector of the ML-based PM damage fault detector models under development. These elements are amplitudes of the frequency components selected in the previous stage of the research: ***X*** *=* [A*_fs_*_+4*fr*_, A*_fs_*_+6*fr*_, A*_fs_*_+10*fr*_, A*_fs_*_+12*fr*_]. However, in the further part of the research, an attempt will also be made to reduce the dimensionality of the input vector, analyzing the impact of individual elements of input vector on the accuracy of the model.

**Figure 11:**
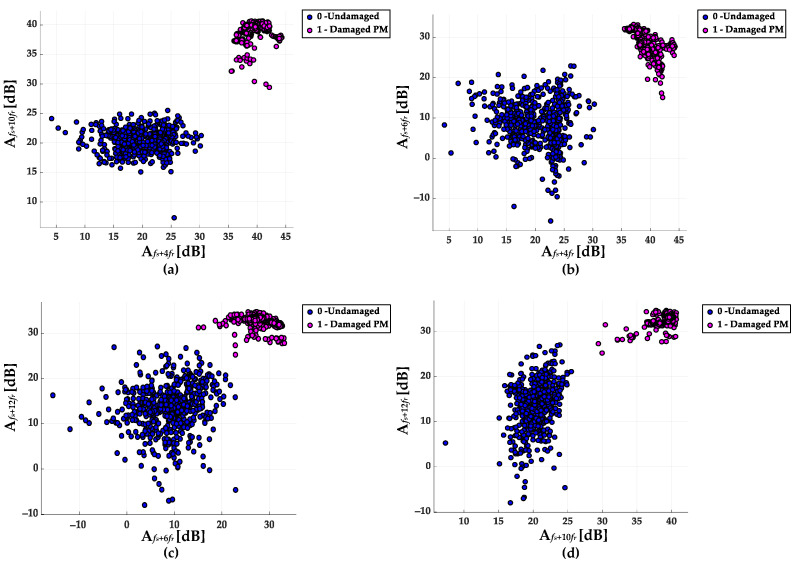
Distribution of the proposed fault features pairs for undamaged and damaged PM (**a**) (A*_fs_*_+4*fr*_, A*_fs_*_+10*fr*_), (**b**) (A*_fs_*_+4*fr*_, A*_fs_*_+6*fr*_), (**c**) (A*_fs_*_+6*fr*_, A*_fs_*_+12*fr*_), (**d**) (A*_fs_*_+10*fr*_, A*_fs_*_+12*fr*_).

The dataset consists of 1080 input vectors. 70% of the input vectors are used in the training process. The remaining 30% are used for offline verification. The vectors that are included in the dataset correspond to the different conditions of the rotor PM magnet: 0 for the undamaged rotor and 1 for the damaged PM, and also the different operating conditions of the analyzed PMSM (*T_L_* = {0; 0.2*T_N_*; 0.4*T_N_*; 0.6*T_N_*; 0.8*T_N_*; *T_N_*}, *f_s_* = {80 Hz, 90 Hz, 100 Hz}). The database was collected during experiments conducted at the test stand described in Section 4. Distribution of the selected pairs of the fault features (amplitudes of the selected harmonics that are elements of the input vector) for the complete dataset are presented in Figure 11. Based on the analysis of this figure, it can be concluded that there is a clear division into the class of undamaged and damaged PM for each of the pair. It can be also seen that the values for damaged PM are much more concentrated.

In the following subsections, the process of training, hyperparameters tuning, and offline verification of the analyzed ML-based PM fault detector models are presented. The accuracy of these models is compared for different parameters. Accuracy defines how often the model’s predictions (responses) are equal to the actual (true) labels. It is defined by the following equation:

(11) $$\mathrm{Accuracy}=\frac{n_{actual}}{N_{t}}\cdot 100\%$$

where *n_actual_* is the number of input vectors that the ML model classified correctly and *N_t_* is the total number of vectors included in the training set.

#### 6.2.1. KNN

**Figure 12:**
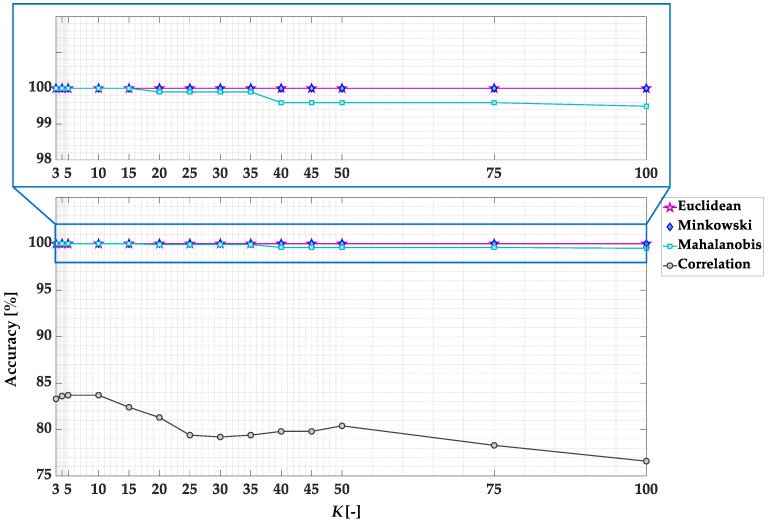
Accuracy of KNN-based PM fault detector model for different *K* values and distance metrics.

In this subsection, the accuracy of the KNN-based PM fault detector is verified for four different distance metrics and a different number of *K* parameters. The accuracies of the classifier models for different parameters are shown in Figure 12 and grouped in Table 3. An accuracy of 100% is achieved for KNN with Euclidean and Minkowski distance metrics in the entire analyzed range of *K* values. In the case of the Mahalanobis distance, 100% accuracy is achieved for *K* values in the range of 3 ÷ 15. The lowest model accuracy is achieved for the correlation distance metric. 

The computational complexity of the algorithm increases along with the increasing value of the *K* parameter. Too low *K* will increase bias and cause misclassifications, leading to underfitting. Therefore, the values of *K* = 1 and *K* = 2 are omitted [43]. Taking into account the computational complexity, to select the best model from those characterized by 100% accuracy, the training times for *K* = 3 and different types of distance metric is compared. The fastest training time (0.847 s) is obtained for KNN with the Euclidean distance metric. For Minkowski and Mahalanobis distances, the times achieved are 0.954 s and 1.001 s, respectively. Based on this detailed analysis, the further tests (off-line and on-line experimental verification) will be conducted for the KNN-based PM damage detector with these parameters.

To reduce the dimensionality of the input vector, the accuracy of the model is verified by successively removing elements of the input vector with the smallest increase as a result of the PM fault (according to the Table 1 and Table 2). For the following input vector: ***X*** *=* [A*_fs_*_+4*fr*_, A*_fs_*_+6*fr*_, A*_fs_*_+10*fr*_] (with A*_fs_*_+12*fr*_ removed), the accuracy of the KNN model is still equal to 100%. Nevertheless, removing A*_fs_*_+4*fr*_ reduced the accuracy to 99.9%, and A*_fs_*_+4*fr*_ to 98.3%. Therefore, the final input vector consists of three elements (A*_fs_*_+4*fr*_, A*_fs_*_+6*fr*_, and A*_fs_*_+10*fr*_).

As it was mentioned in the previous subsection, in the offline tests the remaining 30% (324) of the vectors included in data set are used. To evaluate and compare the effectiveness of the proposed PM damage fault detectors, the detection effectiveness index *D_EFF_* is introduced. It is the ratio of the correctly classified PM states (damaged or undamaged PM) to the number of input vectors. The *D_EFF_* index is defined as follows:

(12) $$D_{EFF}=\frac{Y_{C}}{Y_{C}+Y_{M}}\cdot 100\;\%,$$

where: *Y_C_*—number of correct PM state classifications performed by the model, *Y_M_*—number of PM state misclassifications performed by the model.

**Figure 13:**
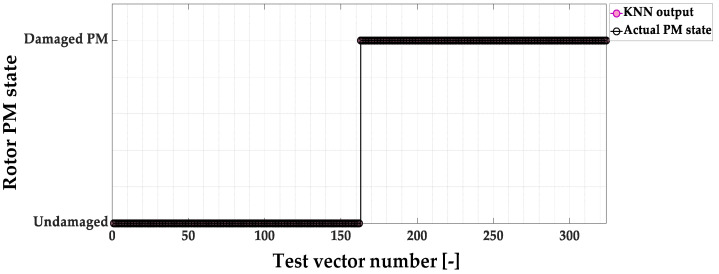
KNN-based PM magnet damage detector responses to the test vectors.

The KNN-based PM fault detector model response to the test data set is shown in Figure 13. The *D_EFF_* value for this test is as high as 100%.

#### 6.2.2. MLP

To select the structure of the MLP models, a constructivist approach is applied. This means that the neurons in the hidden layers are gradually added, and the accuracy of the model is verified for each structure. In the training process, the Levenberg–Marquardt gradient algorithm is applied.

**Figure 14:**
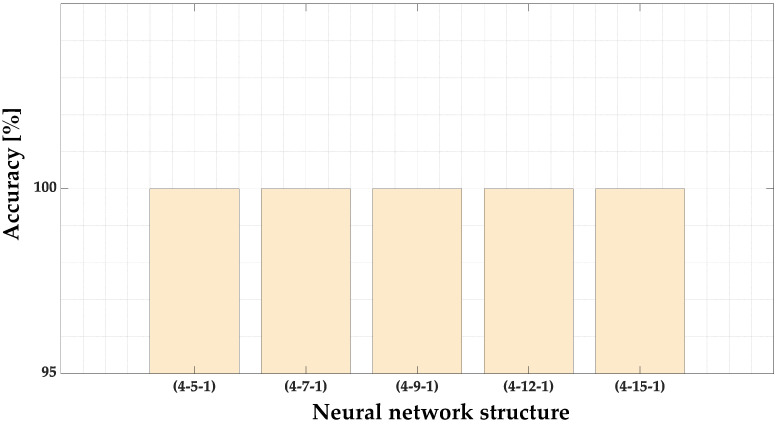
The impact of the MLP structure on the MLP classifier accuracy.

**Figure 15:**
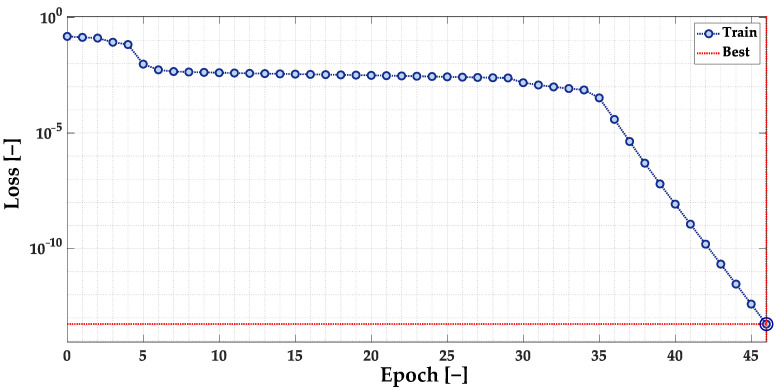
Loss (mean square error) function during the training process of the selected MLP model.

The accuracies of the MLP model for the different network structures are presented in Figure 14 and grouped in Table 4. Based on this comparison, it can be concluded that each of the analyzed structures achieved 100% accuracy of the model. The first of these models (4-5-1) is used in further tests due to its simpler structure. The loss function (mean square error) values during the training process are presented in Figure 15.

As in the case of the KNN model, to reduce the dimensionality of the input vector, the accuracy of this model is verified by successively removing elements of the input vector. For the following input vector: ***X*** *=* [A*_fs_*_+4*fr*_, A*_fs_*_+6*fr*_, A*_fs_*_+10*fr*_] (with A*_fs_*_+12*fr*_ removed) the accuracy of the MLP model is also equal to 100%. The removing of A*_fs_*_+4*fr*_ decreased the accuracy to 99.87%, and A*_fs_*_+4*fr*_ to 99.7%. Therefore, the final input vector consists of the same elements as in the case of the KNN-based detector (A*_fs_*_+4*fr*_, A*_fs_*_+6*fr*_, and A*_fs_*_+10*fr*_).

**Figure 16:**
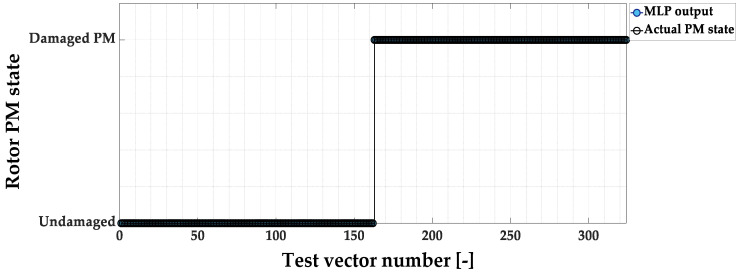
MLP-based PM magnet damage detector responses during to the test vectors.

The responses of the MLP model to the vectors that are included in the test set are shown in Figure 16. The detection effectiveness for this verification is equal to 100%. In the next stage of the research, online tests are conducted to evaluate the developed PM fault detector models.

### 6.3. On-Line Tests of the PM Fault Detectors

**Figure 17:**
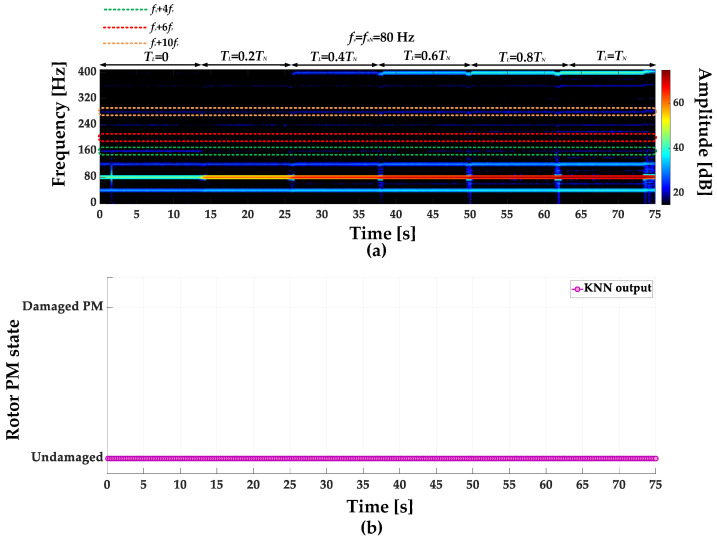
(**a**) Part of the stator phase current STFT spectrogram, (**b**) response of the KNN-based fault detector for undamaged rotor (*T_L_* = var, *f_s_* = 80 Hz).

**Figure 18:**
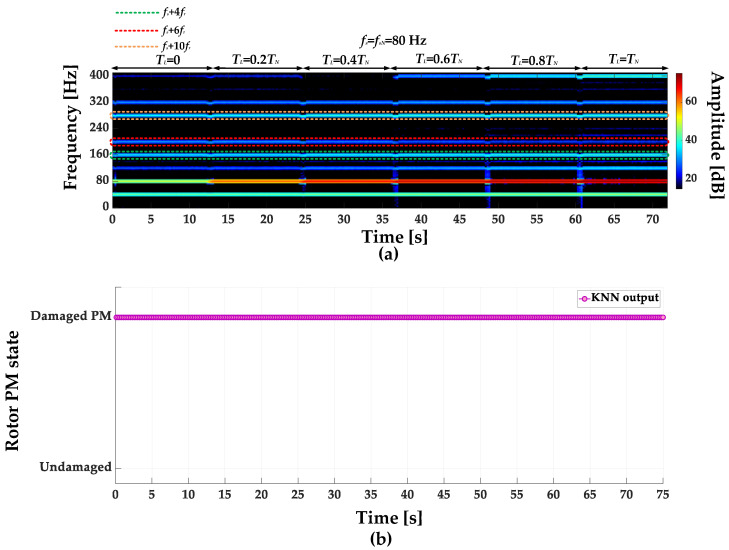
(**a**) Part of the stator phase current STFT spectrogram, (**b**) response of the KNN-based fault detector for damaged PM rotor (*T_L_* = var, *f_s_* = 80 Hz).

**Figure 19:**
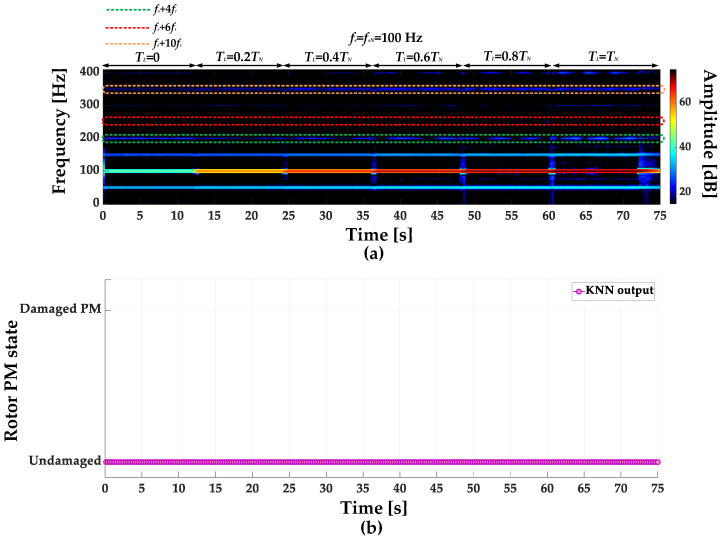
(**a**) Part of the stator phase current STFT spectrogram, (**b**) response of the KNN-based fault detector for undamaged rotor (*T_L_* = var, *f_s_* = *f_sN_* = 100 Hz).

**Figure 20:**
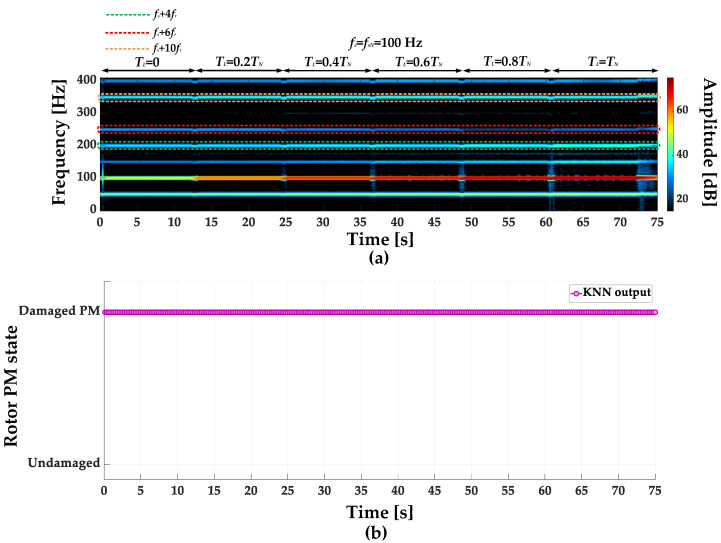
(**a**) Part of the stator phase current STFT spectrogram, (**b**) response of the KNN-based fault detector for damaged PM rotor (*T_L_* = var, *f_s_* = *f_sN_* = 100 Hz).

**Figure 21:**
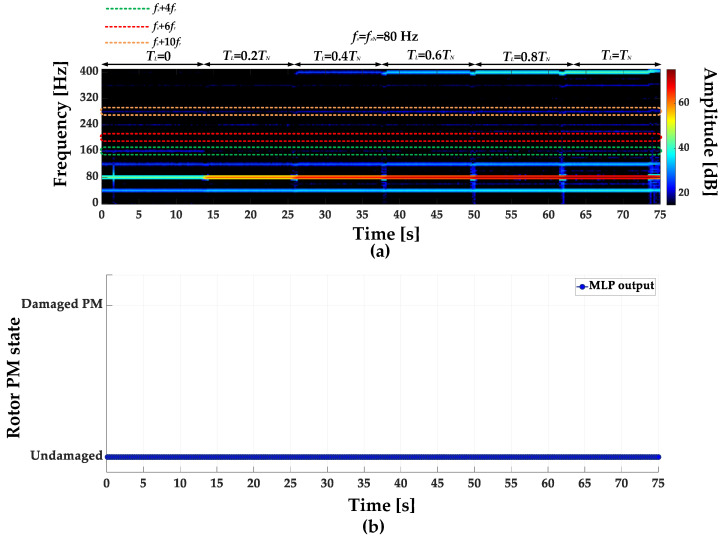
(**a**) Part of the stator phase current STFT spectrogram, (**b**) response of the MLP-based fault detector for undamaged rotor (*T_L_* = var, *f_s_* = 80 Hz).

**Figure 22:**
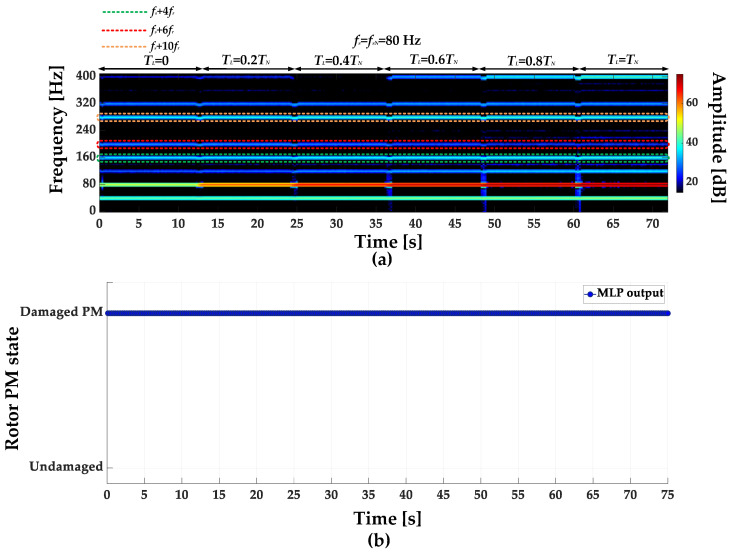
(**a**) Part of the stator phase current STFT spectrogram, (**b**) response of the MLP-based fault detector for damaged PM rotor (*T_L_* = var, *f_s_* = 80 Hz).

**Figure 23:**
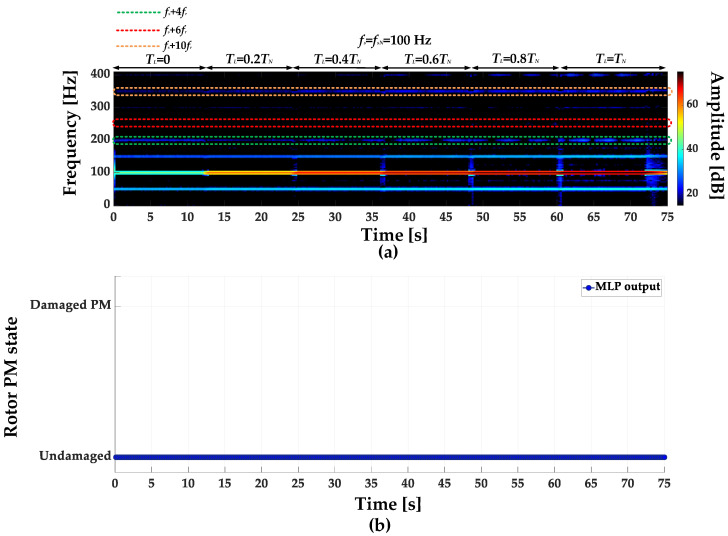
(**a**) Part of the stator phase current STFT spectrogram, (**b**) response of the MLP-based fault detector for undamaged rotor (*T_L_* = var, *f_s_* = *f_sN_* = 100 Hz).

**Figure 24:**
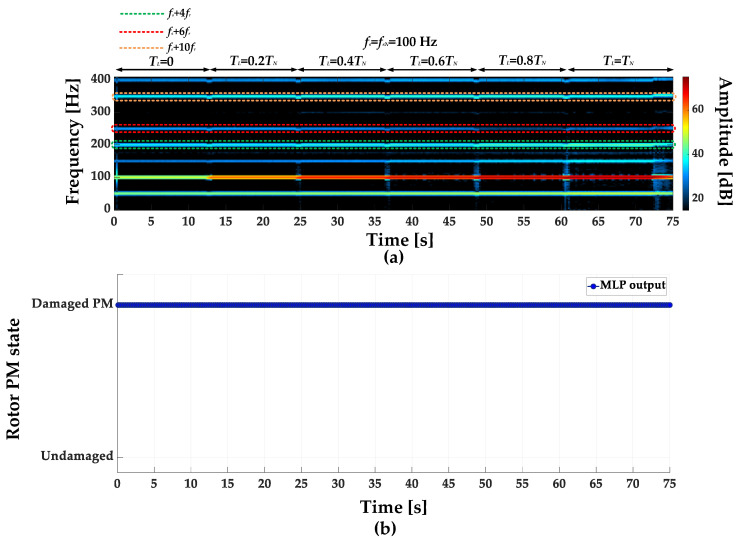
(**a**) Part of the stator phase current STFT spectrogram, (**b**) response of the MLP-based fault detector for damaged PM rotor (*T_L_* = var, *f_s_* = *f_sN_* = 100 Hz).

**Figure 25:**
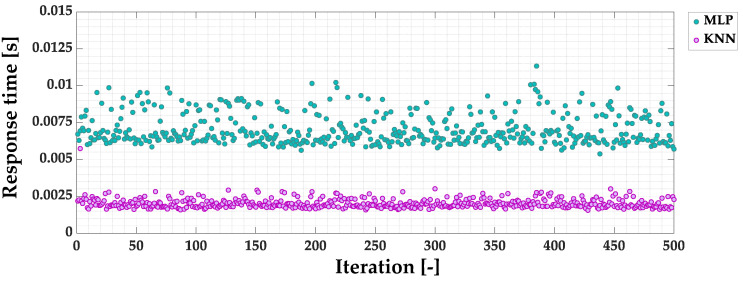
Comparison of the MLP- and KNN-based PM fault detectors response time.

Aiming to the final evaluation of the effectiveness of the developed ML-based PM damage detectors, their detection effectiveness is verified in the online tests. The online operation of the detectors is verified for both undamaged and damaged PM and with successively increased load torque. The load torque is increased with a step of 0.2*T_L_*, up to the rated value *T_N_*. Excerpts from the STFT spectrogram of the stator phase current STFT spectrogram showing changes in the frequency components whose amplitudes are used as the elements of the input vector of the developed models, as well as the responses of the KNN detector operating at *f_s_* = 80 Hz and an undamaged rotor are shown in Figure 17. The dashed frames indicate the frequency of components whose amplitudes are elements of the input vector. In this case, the KNN model correctly classified the condition of the rotor with 100% effectiveness, generating at its output information about the undamaged rotor in the whole range of analyzed load torques. The damaged rotor PM case is shown in Figure 18. The achieved detection effectiveness of the KNN model equals 100%. The results of the same tests but conducted for a nominal power supply frequency (*f_s_* = *f_sN_* = 100 Hz) are shown in Figure 19 and Figure 20. While the results of the MLP-based PM damage detector for the same cases are presented in Figure 21, Figure 22, Figure 23, Figure 24 and Figure 25. For the MLP model, the *D_EFF_* in all cases equals 100%. This confirms the high performance of the STFT analysis in the extraction of PM damage symptoms and a very good choice of the elements of the model input vector.

### 6.4. Summary

The use of the STFT analysis of the stator phase current signal and a thorough analysis of the increases in harmonic amplitudes made it possible to select the components most sensitive to PM damage. The selection of the input vector elements of the applied ML models allowed the achievement of 100% accuracy of the KNN and MLP models, as well as *D_EFF_* = 100% during off-line and on-line tests. To choose the best of the two discussed ML models, their response times are compared. In Figure 25 the comparison of the MLP- and KNN-based PM fault detectors response time for 500 iterations is presented. In the case of the KNN model, the response time is about three times shorter compared to the MLP detector. The average response time for the KNN model equals 0.0020 s, whereas for the MLP model it is 0.0071 s. The comparison of the details of the analyzed models are grouped in Table 5.

## 7. Conclusions

In this paper, an effective PMSM demagnetization fault detection method based on STFT analysis of the stator phase current and ML-based models is proposed. The presented experimental verification results confirm the applicability of STFT analysis in the process of the extracting rotor PM damage symptoms in PMSM drives.

Careful selection of the input vector elements of the analyzed ML models: KNN and MLP, resulted in 100% model accuracy and 100% effectiveness in off-line and on-line tests of these detectors. The significant impact of the choices of these elements, as well as the key parameters of the ML models on their effectiveness were also analyzed and confirmed.

Among the most important conclusions arising from the analysis of the results of the presented research is that with an efficient symptom extraction stage, it is not necessary to use more advanced ML algorithms such as neural networks, including those with a deep structure, but simple ML algorithms such as KNN can be used. This allowed not only the achievement of high effectiveness, but also very short training and response time (0.0020 s), three times shorter compared to the MLP model (0.0071 s).

Taking advantage of the simplicity of the KNN algorithm and its high effectiveness in PM fault detection, further research will focus on the embedded (microcontroller) implementation of the fault diagnosis system that will be based on the proposed methodology. In addition, the effect of measurement accuracy: sampling frequency, noise effects, on-detection effectiveness will also be considered in the scope of future research.

## Figures and Tables

**Table 1:** The analysis of amplitude increases of the selected frequency components characteristic of the PM fault for different load torques.

Failure Frequency *f_PMDamage_*	*T_L_* [p.u]						A_DIFFAvg_ [dB]	σ [dB]
	0	0.2*T_N_*	0.4*T_N_*	0.6*T_N_*	0.8*T_N_*	*T_N_*		
	*A_DIFF_* [dB]	*A_DIFF_* [dB]	*A_DIFF_* [dB]	*A_DIFF_* [dB]	*A_DIFF_* [dB]	*A_DIFF_* [dB]		
*f_s_* – 2*f_r_*	11.30	10.45	10.72	10.05	9.69	9.66	10.31	0.64
*f_s_* + 2*f_r_*	1.90	2.60	1.50	1.30	2.30	2.70	2.05	0.58
***f_s_* + 4*f_r_***	**15.10**	**18.30**	**17.80**	**16.08**	**16.50**	**16.40**	**16.70**	**1.17**
***f_s_* + 6*f_r_***	**23.20**	**23.55**	**21.50**	**19.77**	**14.10**	**18.90**	**20.17**	**3.49**
***f_s_* + 10*f_r_***	**19.23**	**19.80**	**18.80**	**18.30**	**18.70**	**18.00**	**18.81**	**0.65**
***f_s_* + 12*f_r_***	**14.87**	**17.63**	**15.77**	**15.30**	**9.96**	**9.70**	**13.87**	**3.27**
*f_s_* + 18*f_r_*	17.35	14.40	12.40	4.05	2.83	2.58	8.94	6.55
*f_s_* + 22*f_r_*	10.20	9.52	11.36	14.20	15.96	14.00	12.54	2.55

**Table 2:** The analysis of amplitude increases of the selected frequency components characteristic of the PM fault for different power supply frequency.

Failure frequency *f_PMDamage_*	*f_s_* [Hz]					*A_DIFFAvg_* [dB]	*σ* [dB]
	60	70	80	90	100		
	*A_DIFF_* [dB]	*A_DIFF_* [dB]	*A_DIFF_* [dB]	*A_DIFF_* [dB]	*A_DIFF_* [dB]		
*f_s_* − 2*f_r_*	4.57	7.53	8.34	8.88	9.66	7.80	1.96
*f_s_* + 2*f_r_*	1.69	3.51	2.06	5.70	2.70	3.13	1.59
***f_s_* + 4*f_r_***	**18.00**	**15.13**	**18.69**	**14.57**	**16.40**	**16.56**	**1.78**
***f_s_* + 6*f_r_***	**14.40**	**6.47**	**19.28**	**17.00**	**18.90**	**15.21**	**5.25**
***f_s_* + 10*f_r_***	**17.90**	**18.60**	**18.71**	**19.20**	**18.00**	**18.48**	**0.54**
***f_s_* + 12*f_r_***	**14.08**	**17.81**	**16.04**	**12.64**	**9.70**	**14.05**	**3.12**
*f_s_* + 18*f_r_*	3.18	0.38	0.80	1.90	2.58	1.77	1.18
*f_s_* + 22*f_r_*	12.05	11.92	8.11	12.07	14.00	11.63	2.15

**Table 3:** The KNN classifier accuracies for different key parameters.

*K* [-]	Distance Metric			
	Euclidean	Minkowski	Mahalanobis	Correlation
3	100.0%	100.0%	100.0%	83.3%
4	100.0%	100.0%	100.0%	83.6%
5	100.0%	100.0%	100.0%	83.7%
10	100.0%	100.0%	100.0%	83.7%
15	100.0%	100.0%	100.0%	82.4%
20	100.0%	100.0%	99.9%	81.3%
25	100.0%	100.0%	99.9%	79.4%
30	100.0%	100.0%	99.9%	79.2%
35	100.0%	100.0%	99.9%	79.4%
40	100.0%	100.0%	99.6%	79.8%
45	100.0%	100.0%	99.6%	79.8%
50	100.0%	100.0%	99.6%	80.4%
75	100.0%	100.0%	99.6%	78.3%
100	100.0%	100.0%	99.5%	76.6%

**Table 4:** The MLP classifier accuracies for different structure of the network.

MLP Structure	Accuracy	
(4-5-1)	100.0%	
(4-7-1)	100.0%	
(4-9-1)	100.0%	
(4-12-1)	100.0%	
(4-15-1)	100.0%	

**Table 5:** The comparison of the analyzed ML-based PM faul detectors details.

	ML-Based PM Fault Detector	
	KNN	MLP
Accuracy [%]	100.0	100.0
Offline test *D_EFF_* [%]	100.0	100.0
Online tests *D_EFF_* [%]	100.0	100.0
Response time [s]	0.0020	0.0071

## Data Availability

Not applicable.

## Appendix A

**Table A1:** Rated parameters of the tested PMSM.

Name of the Parameter	Symbol	Units	
Power	*P_N_*	2500	[W]
Torque	*T_N_*	16	[Nm]
Speed	*n_N_*	1500	[r/min]
Stator phase voltage	*U_sN_*	325	V
Stator current	*I_sN_*	6.6	[A]
Frequency	*f_sN_*	100	[Hz]
Pole pairs number	*p_p_*	4	[-]
Number of stator turns	*N_st_*	2 × 125	[-]
